# Cone-Beam Computed Tomography for Evaluation of Apical Transportation in Root Canals Prepared by Two Rotary Systems

**Published:** 2014-03-08

**Authors:** Zahra Sadat Madani, Azam Haddadi, Sina Haghanifar, Ali Bijani

**Affiliations:** a * Department of Endodontics, Dental School, Babol University of Medical Sciences, Babol, Iran;*; b * Department of Endodontics Dental school Mazandaran University of Medical Sciences, Sari, Iran; *; c * Department of Oral and Maxillofacial Radiology, Dental School, Babol University of Medical Sciences, Babol, Iran;*; d *Non-Communicable Pediatric Diseases Research Center, Babol University of Medical Sciences, Babol, Iran*

**Keywords:** Apical Transportation, CBCT, Cone-Beam Computed Tomography, Imaging, Root Canal Therapy

## Abstract

**Introduction:** Due to the importance of apical transportation during root canal preparation, the aim of the current study was to use cone-beam computed tomography (CBCT) to assess the extent of apical transportation caused by ProTaper and Mtwo files. **Methods and Materials:** Forty extracted maxillary first molars with 19-22 mm length and 20-40 degrees of curvature were selected. The mesiobuccal canals were prepared using either Mtwo or ProTaper rotary files (*n*=20). CBCT images were obtained before and after canal preparation to compare the apical transportation in different cross-sections of mesial and distal surfaces. The apical transportation values were analyzed using the SPSS software. The results were compared with student’s t-test and Mann–Whitney U test. **Results:** There was no significant difference in the extent of apical transportation between Mtwo and ProTaper systems in different canal cross-sections. The apical transportation value was less than 0.1 mm in most of the specimens, which was clinically acceptable. **Conclusion:** Considering the insignificant difference between the two systems, it can be concluded that both system have low rates of apical transportation and can be assuredly used in clinical settings.

## Introduction

Mechanical preparation of the root canal system is one of the most important steps in root canal treatment. The aim of biomechanical cleaning of the root canal is the removal of its contents (especially microorganisms). This aim can be achieved through enlargement and shaping of the canal and also its chemical debridement, while maintaining the radicular anatomy [1]. Although, various techniques have been developed to prevent procedural mishaps during root canal preparation [2]; there are still some difficulties in complete canal preparation especially for curved and flattened ones [3, 4]. Canal transportation happens when more amounts of dentin is removed from the outer walls of the areas apical to the canal curvature and inner walls of the areas coronal to curvature; as a result one side of the canal in a single direction is removed more compared to other equidistant directions from the main tooth axis [5]. Canal transportation results in the displacement of the physiologic end of the canal to a new operator-made location on the external surface of the root, leading to accumulation of the residual debris and microorganisms due to the absence of proper and adequate debridement of the root canal [4, 6].

The shape created due to canal transportation does not provide a resistant form to condense gutta-percha and results in its poor compaction and over-extension of root canal fillings, which finally leads to failure of the treatment [7].

Most rotary nickel-titanium (NiTi) files are used based on crown-down technique in which the preparation is done by using the file sizes in a descending order until the apex is reached. Mtwo system (VDW, Munich, Germany) is an exception in which the first file is used to full working length (*aka.* single length technique) by the practitioner. Moreover, Mtwo is one of the systems with small-sized files such as 10/0.04 and 15/0.05 being used to reach the apical third at the beginning of canal preparation [8]. Using this system, the root canal anatomy is claimed to be kept unchanged [9]. It is claimed to have less file breakage and faster running speed. Besides, compared with other systems, it significantly removes more debris [10, 11].

ProTaper system (Dentsply Maillefer, Ballaigues, Switzerland) is also being widely used for canal preparation. The files of this system are specially designed which makes them appropriate for preparation of highly calcified and curved root canals [12].

Recently, noninvasive techniques are developed to evaluate the canal anatomy and to compare the canal shape before and after preparation. Using computed tomography (CT), proper cross-sections of roots can be provided and 3-dimensional CT images can be reconstructed simultaneously [13, 14]. Cone-beam computed tomography (CBCT) is more precise compared with other routine techniques. It does not require destruction of specimen. It is also highly reproducible and several images of the canals can be captured [5, 14-16].

The aim of this *in vitro* study was to use CBCT images to compare the apical transportation after canal preparation with either of the rotary systems, *i.e.* ProTaper and Mtwo.

## Methods and Materials

The research protocol was approved by the Ethics committee of Babol University of Medical Sciences, Babol, Iran (Grant no. 6830). In this *in vitro* study, 40 extracted human maxillary first molars with fully developed apices were obtained with the length of mesiobuccal (MB) canal being in the range of 19-22 mm and canal curvature of 20-40 degrees, according to the Schneider’s method [17]. The teeth were kept in 0.1% thymol solution at 9° C for disinfection, and 24 h before use, they were washed with running tap water to eliminate traces of thymol and were then stored in normal saline at 4° C until further processing.

Initially, the access cavities were prepared and the MB canals were localized. Afterwards, the MB canal was explored with a size #10 K-file (Mani, Tochigi, Japan) until the file tip could be visible from the apex. Then the working length was calculated by reducing 1 mm from this length. The teeth were embedded in acrylic resin blocks to facilitate the imaging process and maintain reproducibility of the CBCT images.

Before starting canal preparation, 3-dimensional, high resolution CBCT images were obtained using Cranex 3+ (Soredex, Helsinki, Finland) with 8.6 cm field of view (FOV). The 0.5 mm-layer of images were taken axially, with 3-mm distance from the radiographic apex, and perpendicular to the long axis of the root. The pre-procedural images were recorded to be later compared with post-preparation images.

Then, the teeth were randomly assigned to 2 equal groups (*n*=20). In both groups, direct access was provided using the crown-down technique with sizes 3 to 1 Gates-Glidden drills (Dentsply, Maillefer, Switzerland). A #10 K-file was also used with RC-prep (Premier Dental Products, Philadelphia, USA) to provide a glide-path and then canals were flushed with saline and filled with 2 mL of 2.5% NaOCl in both groups.

***Group 1:***

In this group, the canals were cleaned and shaped using Mtwo file system (VDW, Munich, Germany) and a handpiece installed on an electric motor (Endo-Mate TC, NSK, Nakanishi Inc., Tokyo, Japan) used at 300 rmp and a torque of 3 Nm according to the manufacturer’s instruction. Apical preparation was performed with file sizes 10/0.04, 15/0.05, 20/0.06 and 25/0.06. Each file was discarded after being for once.

***Group 2:***

In this group, the canals were prepared using ProTaper system (Dentsply Maillefer, Ballaigues, Switzerland) and a handpiece installed on an electric motor used with 300 rmp and 3 Nm torque according to the manufacturer’s instructions. Apical preparation was performed with file sizes S1, S2, F1 and F2 (25/0.06). Each file was discarded after being used for once.

In both groups, all preparation stages were done by a single operator. According to the study protocol, the operator had to work on only five canals per day at a certain time throughout the day. It helped to apply the same amount of force for canal preparation and to eliminate the effect of operator’s fatigue on the results.

After finishing the preparation stages, post-instrumentation CBCT images were obtained exactly similar to what had been done, before preparation. Using the Adobe Photoshop (Version 7, Adobe Systems Inc., San Jose, CA) for evaluating the apical transportation, we applied the technique proposed by Gambill and colleagues [16, 18]. According to this technique, the following distances were considered:

*A1:* Minimal distance of the external side of the root section from the external mesial edge of the uncleaned canal.

*B1:* Minimal distance of the external side of the root section from the external distal edge of the uncleaned canal.

*A2:* Minimal distance of the external side of the root section from the external mesial edge of the cleaned canal.

*B2:* Minimal distance of the external side of the root section from the external distal edge of the cleaned canal.

Finally the amount of canal transportation was calculated using the following formula: [(A1-A2)–(B1-B2)]. According to this formula, a result of “zero” showed no canal transportation; while, any other result indicated that transportation had occurred in the canal. Apical transportation values were determined using both systems at the levels of 2, 2.5, and 3 mm from the apex to the mesial and distal surfaces. The values were analyzed using SPSS software, version 18. The student’s t-test and Mann–Whitney U test were used to compare the data and the significance level was set at 0.05.

**Table 1:** Mean (SD) of apical transportation on different cross-section in ProTaper and Mtwo systems

**Distance from apex (mm)**	**System Mean (SD)**		***P-value *** **(t-test)**	**Power**	***P-value *** **(Mann–Whitney U)**
	**ProTaper **	**Mtwo **			
**2 mm (mesial surface)**	0.111 (0.167)	0.09 (0.121)	0.659	0.0741	0.904
**2 mm from the apex on distal surface**	0.06 (0.125)	0.058 (0.085)	0.930	0.0504	0.355
**2.5 mm from the apex on mesial surface**	0.149 (0.211)	0.083 (0.096)	0.215	0.2468	0.231
**2.5 mm from the apex on distal surface**	0.07 (0.175)	0.072 (0.073)	0.963	0.0503	0.289
**3 mm from the apex on mesial surface**	0.143 (0.156)	0.094 (0.095)	0.233	0.2244	0.289
**3 mm from the apex on distal surface**	0.099 (0.191)	0.096 (0.109)	0.952	0.0504	0.242

## Results

Table 1 shows the mean apical transportation in each group. The apical transportation in the ProTaper system was slightly more than that of Mtwo system but there was no significant difference between Mtwo and ProTaper file systems in different cross-sections of the canal regarding the amount of apical transportation (*P*>0.05).

## Discussion

Using the double radiographic superimposition technique, two dimensional radiographs can be adjusted and mesiodistal and buccolingual views of radiographs can be assessed and the extent of apical transportation can be determined. Actually, none 

of the specimens showed their maximum curvature degree in the mentioned plans. Therefore, real values of canal transportation cannot be measured under these conditions [19].

CBCT has been frequently used in endodontic treatments due to the utilization of Photoshop software [5]. The technique is more accurate than conventional methods, and the results are highly reliable and multiple images can be obtained from the canals. Also by using CBCT images, complete information can be taken from the root canal before, during and after the mechanical preparation [5, 20]. Considering the advantages of CBCT imaging, this technique was used for evaluation of apical transportation in the present study.

Results of the present study indicated that the amount of apical transportation was less than 0.1 mm at all sections using both ProTaper and Mtwo file systems. There was no significant difference between the two systems in this regard. The apical transportation in the ProTaper system was slightly more than that of Mtwo system especially at the levels of 2.5 and 3 mm of the apex from the mesial surface; although this difference was not statistically significant. Therefore, regardless of this small amount of transportation, both systems had the capability to maintain the canal centralization and could be used in clinical settings. Peters *et al*. reported that apical transportation ≤0.1 mm is clinically acceptable [21]. In the present study, this value was less than 0.1 mm in most cases; although in some cases it was more. It seems that similarities in apical transportation in various rotary systems are related to the amount of cervical preparation.

Apical transportation may be attributed to some factors such as the preparation technique, physical properties of alloys, and design of the instrument [22]. According to Iqbal and colleagues, small degrees of transportation are associated with the ability of the file to remain centered within the canal, which in turn depends on the physical properties of used alloys as well as the sharp and cone shape of the instrument’s tip [23].

Kuzekanani and colleagues showed that canal transportation in Mtwo system was notably less than the ProTaper system. They also claimed that because of the least changes in canal curvature and maintaining the original shape of the root canal, Mtwo system was better than ProTaper [24]. Although the present study revealed similar results, the difference between the two systems was not significant.

Khalilak *et al*. reported that Mtwo files had less canal-centering ability compared to ProFile and RaCe [25]. In another study, Javaheri and colleagues proposed that more flexible instruments with less taper like RaCe should be used for preparation of curved canals [26]. Also, Nagaraja and colleagues assessed canal transportation, centering ability, and the amount of remaining dentine thickness after instrumentation using manual NiTi K-file and ProTaper rotary system. They concluded that because ProTaper files could cause greater extent of canal transportation, they should be used cautiously especially in curved canals [18].

Moreover, Özer evaluated root canal transportation after using various rotary systems using computed tomography and found insignificant apical transportation in all such systems [27]. In that report, despite using non-cutting tips in all the rotary systems, some degrees of apical transportation were reported. Therefore, it seems that such incidence is inevitable in all rotary and manual systems. In another study, Veltri and colleagues reported that preparation of curved canals with ProTaper system is acceptable and is associated with minimal canal transportation [28].

On the other hand, other studies have reported specific degrees of transportation after using the ProTaper system [24, 29]. Yoshimine *et al*. evaluated the canal transportation after preparation of S-shape canals using RaCe, ProTaper, and K3 systems. They found more deviation from the original canal path using ProTaper system than RaCe and K3 systems [29].

## Conclusion

Considering the absence of significant differences in apical transportation values following preparation with Mtwo and ProTaper file systems, it can be concluded that both systems have similar canal-centering ability, canal transportation and similar ability to maintain the shape of the canal after preparation. Therefore, both systems can be used in clinical settings with minimal apical transportation.
